# Simulation of the Kinematic Condition of Radial Shear Rolling and Estimation of Its Influence on a Titanium Billet Microstructure

**DOI:** 10.3390/ma15227980

**Published:** 2022-11-11

**Authors:** Mikhail M. Skripalenko, Boris V. Karpov, Stanislav O. Rogachev, Liudmila M. Kaputkina, Boris A. Romantsev, Mikhail N. Skripalenko, Tran Ba Huy, Viktor A. Fadeev, Andrei V. Danilin, Yuri A. Gladkov

**Affiliations:** 1 Department of Metal Forming, National University of Science and Technology MISiS, Leninski Prospect 4, 119049 Moscow, Russia; 2 Nauchno-Proizvodstvennyj Centr Obrabotka Metallov Davleniem Intitutskiy Proezd, 2, Zavoda Mosrentgen, 142771 Moscow, Russia; 3 Department of Physical Metallurgy and Physics of Strength, National University of Science and Technology MISiS, Leninskiy Prospekt 4, 119049 Moscow, Russia; 4 Viettel Aerospace Institute, Hanoi 13112, Vietnam; 5 Metal Forming Department, Bauman Moscow State Technical University, Vtoraya Baumanskaya Street, 5/22, 105005 Moscow, Russia; 6 QuantorForm LLC, 123308 Moscow, Russia

**Keywords:** radial shear rolling, velocity of metal flow, acceleration, slowing, accumulated strain, grain size, total velocity, neutral surface

## Abstract

The finite element method (FEM) computer simulation of the three-high radial shear rolling of Ti-6Al-4V alloy round billets was conducted using QForm software. The simulation was performed for the MISIS-100T rolling mill’s three passes according to the following rolling route: 76 mm (the initial billet diameter) →65 mm→55 mm→48 mm (the final billet diameter). The change in the total velocity values for the points on the radius of the 48 mm diameter billet was estimated while passing the rolls’ draft. The relative increase in the accumulated strain was estimated for the same points. Then, experimental shear rolling was performed. Grain sizes of the α- and β-phases were estimated in the cross section of the final billet at the stationary stage of rolling. The grain size distribution histograms for different phases were plotted. An area was found in the billet’s cross section in which the trend of change in the total velocity of the points changed. This area represented a neutral layer between the slowing peripheral segments of the billet and the accelerating central segments of the billet. Inside this neutral layer, the limits of the cylindrical surface radius value were estimated. Experimental radial shear rolling was performed to compare the experimental rolling results (the billet microstructure investigation) with the computer simulation results. The computer simulation obtained two estimations of the radius limits: 8–16 mm (based on the analysis of the total velocity change) and 12–16 mm (based on the accumulated strain’s relative increment change). The experimental rolling obtained two more estimations of the radius limits: 8.4–19.5 mm and 11.3–19.7 mm—based on the results of the microstructure investigation. It was confirmed that varying the kinematic and deformation parameters of radial shear rolling allows regulation of the thickness of the peripheral fine-grain layer and the diameter of the central coarse-grain layer of the rolled billets.

## 1. Introduction

Radial shear rolling (RSR) is a screw (or rotary or helical) rolling process when the feed angle of the rolls is 18–20 degrees or more [1,2]. The trajectory-controlled variant is the most widespread variant of RSR [2,3].

RSR enables rolling almost all deformable alloys with intensive grain structure refinement and the complex improvement of properties. This is due to the unique kinematic conditions of the helical metal flow. The deformation zone, at the stationary stage, consists of two zones where the changes in the metal flow velocity are opposite. The peripheral layers of the billet slow while rolling, and the central layers accelerate. There is a neutral layer between them, and the total velocity remains almost unchanged inside this neutral layer. As a result, intensive macro- and micro-shear strains are activated, and a gradient structure with a fine-grained outer layer and a coarse-grained central zone is formed. This kinematic feature was firstly detected and analytically described in [2,3]. The screw-rolling technique based on this kinematic feature was patented [4] and successfully used for different deformation purposes and obtaining the structured condition of metals and alloys for long volumes (the long round billets) [5]. The main deformation and technological capabilities of the RSR are directly connected to the specified kinematic condition. It is the same as the connection of two-high screw piercing with the so-called Mannesmann effect. 

RSR-related research is mostly dedicated to the formation of the structure and properties of the round billets of different materials: titanium and its alloys [6,7,8,9], copper and its alloys [10,11], magnesium alloys [12,13,14,15,16], nickel alloys [17], zirconium alloys [18], aluminum and its alloys [19,20], and steels of different chemical compositions for various applications [21,22,23,24,25,26]. The combination of RSR with other metal-forming techniques is effective in terms of distinctive features formation. For instance, it is effective for the formation of the super-elastic properties of Ti-18Zr-14Nb alloys, which are used for orthopedic implants [27]. Computer simulation, including FEM-based, is applied for the estimation of the stress–strain state parameters and temperature distribution through the rolled billet’s volume during RSR [28,29,30,31,32]. Some research has been dedicated to the joint use of computer simulation and experimental RSR for different purposes: the prediction of a possible billet’s fracture during RSR [11], the estimation of the features of the stress–strain state parameters’ distribution during the stationary and nonstationary stages of RSR [33,34,35], research of the billet’s microstructure and property formation [36,37,38,39], and the estimation of the stress–strain state parameters’ distribution in the deformation zone during RSR [14,20,40,41]. It has been noted [11,35] that further investigations of three-high screw rolling are necessary because the billet’s stress–strain state features and their influence on the billet’s microstructure have not been completely detected. For this, the joint use of computer simulation and experiments are effective. One of the key features, due to the very specific stress–strain state both for three-high screw rolling and the three-high RSR process, is the presence of the so-called ring-shaped area or ring-shaped zone in the cross section of the billet. The presence of this ring-shaped area makes the billet prone to ring-shaped fracture [42]. The results of [11,34,35] revealed a ring-shaped area with minimal hardness values. Using different damage criteria in some cases is effective to reveal the presence of this ring-shaped area [43]; in some cases, it is not [11]. The technique presented in [11] enabled identification of the ring-shaped area, but the technique was very time consuming and was not verified for other RSR regimes or billet materials. It seems that the ring-shaped area may be considered a neutral layer dividing the peripheral and central zones of the billet. It is important to develop a technique to estimate the size of this neutral layer using both the stress–strain state and the kinematic parameters’ distribution along with the microstructure features. It is also relevant that the RSR parameters directly influence the microstructure, considering the results in [44,45].

The objective of this research is the computer simulation of the RSR kinematic conditions and the stress–strain state features using QForm software and the microstructure analysis of the Ti-6Al-4V alloy billets rolled using an MISIS-100T three-high screw rolling mill to estimate the billet’s neutral layer dimensions.

## 2. Materials and Methods

### 2.1. Computer Simulation Technique

**Figure 1:**
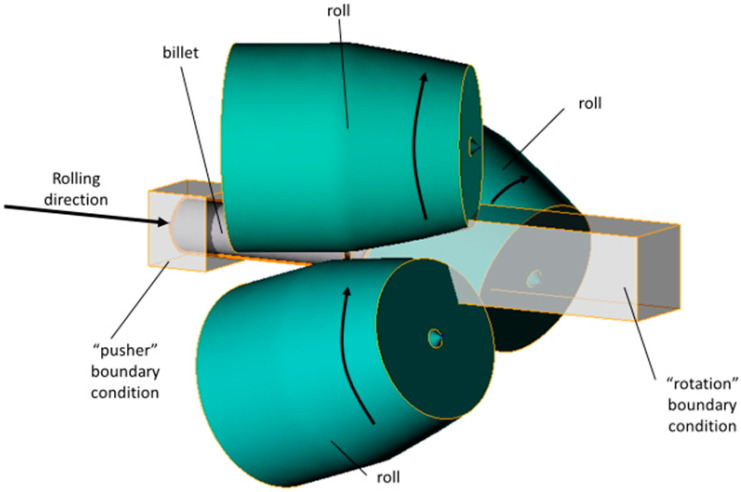
QForm model for computer simulation of three-high radial shear rolling in an MISIS-100T rolling mill (arrows identify rolls’ rotation directions).

**Figure 2:**
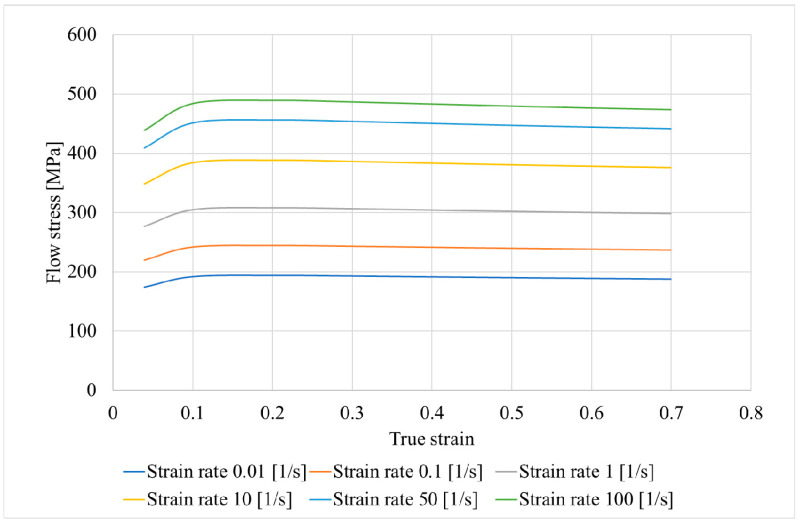
Flow stress of the Ti-6Al-4V alloy at 900 °C for different strain rates.

For the computer simulation, models of rolls and billet were made using SolidWorks; then, the assembly was conducted from the models, saved in “.step” format, and uploaded to the QShape geometry editor. The diameter of the rolls in the gap (or draft) was 300 mm, the cupped scheme of roll positioning was used, and the rolls’ exit taper was 11 degrees. The finite element mesh was generated for each object of the assembly, and the roll rotation axes were set using the QShape menu tools. After that, the assembly was uploaded in QForm, the initial and boundary conditions were set. The “pusher” and “rotation” boundary conditions were set for the billet (Figure 1). The friction factor was set at 5 in the Siebel friction law for “roll–billet” interaction. The rolls’ rotational velocity was set 9.42 rad/s. The feed angle of the rolls was set at 20 degrees, and the inclination angle of rolls was set at 10 degrees. The simulation was conducted in accordance with the condition that a maximum tetrahedral finite element size was not more than 2 mm. The billet’s material was set as Ti-6Al-4V, and the flow stress of this material at different strain rates and temperatures was given in table form. The heat exchange between the rolls and billet was set as “Simple” [46]. After that, the QForm preprocessor, using the table data, obtained the flow stress curves using interpolation. The flow stress depended on the strain (Figure 2) at different temperatures (750, 800, 900, 1000, and 1100 °C) and strain rates (0.01, 0.1, 1, 10, 50, and 100 s^−1^). The RSR was simulated according to the route: Ø76→Ø65→Ø55→Ø48 mm.

**Figure 3:**
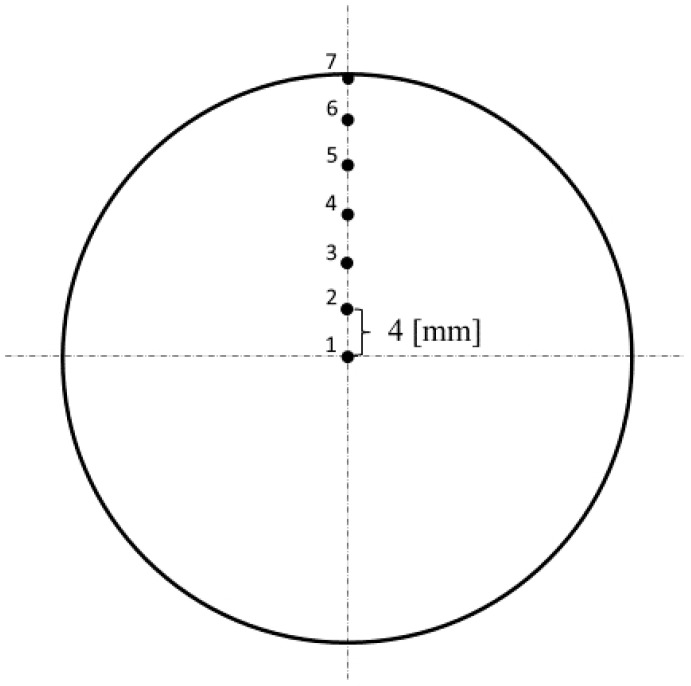
Points on the radius in the cross section of the billet at the stationary stage; the total velocity and accumulated strain calculation was made for these points using QForm software.

When the computer simulation was complete, 7 points with 4 mm equal distance between them were chosen in the cross section of the 48 mm diameter billet at the stationary stage. One of the points was in the center of the billet, and one was on the surface (Figure 3). The accumulated strain and the total velocity values were calculated for these points. 

### 2.2. Experimental Rolling Technique 

**Figure 4:**
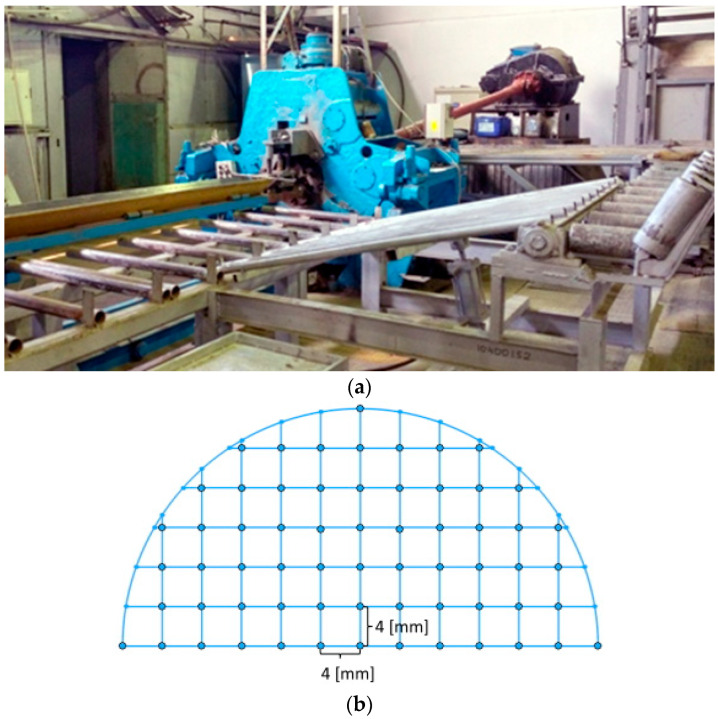
MISIS-100T mill (**a**) and visual field location scheme for the metallographic analysis of the cross section (**b**).

The RSR of the Ti-6Al-4V alloy billets was performed using an MISIS-100T three-high mill (Figure 4a) in three passes according to the route Ø76→Ø65→Ø55→Ø48 mm. The feed angle of the rolls was 20 degrees, and the inclination angle of the rolls was 10 degrees. Before rolling, the billet was heated to 940–950 °C for 1 h. The billet was heated between passes to the rolling temperature for 5–10 min. The rolls rotated with a speed equal to 9.42 rad/s. When the rolling was over, the billet was air-cooled. A semicircle specimen was cut from the middle of the 48 mm diameter billet (final billet) to investigate the microstructure. The cross section was created for microstructure investigation. The microstructure of the Ti-6Al-4V alloy specimen was studied using a Micromet 5101 Buehler optical microscope at ×100 and ×500 magnifications. The visual field location scheme of the metallographic analysis of the cross section is presented in Figure 4b.

The cross-section surface was etched by an agent with the following composition: 30 mL H_2_O + 30 mL HNO_3_ + 30 mL H_2_SO_4_ + 10 mL HF. The chemical composition of the alloy billet is presented in Table 1.

## 3. Results

**Figure 5:**
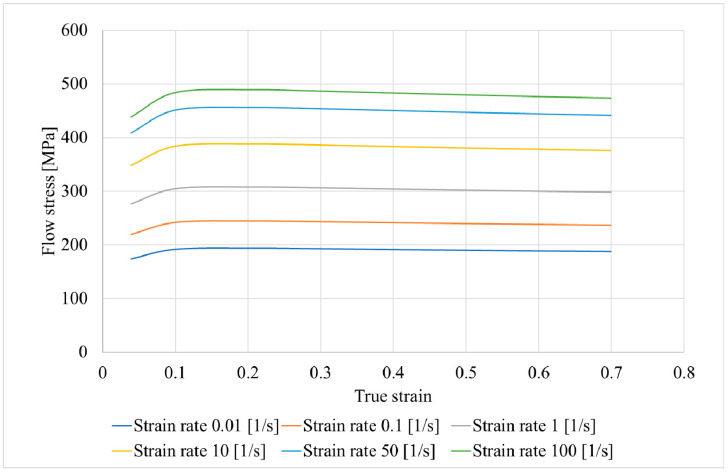
Total velocity change of the tracked points during the third pass (the numbers near the curves correspond to the points in Figure 3).

The computer simulation results allowed estimation of the variation with time of the total velocity for the chosen points (Figure 5). The total velocity of the points near the surface (points 5, 6, 7) decreased during each of the passes, while the total velocity of the points near to the center increased; the third (final) pass is shown in Figure 5. 

From Figure 5, the billet can be divided into two regions: the region with a decrease in the total velocity (peripheral region of the billet) and the region with an increase in the total velocity (central region of the billet). The trend of the total velocity changed for all the passes between points 3 and 5 (Figure 3). Hence, it can be deduced that there was a neutral cylindrical layer [4] containing a neutral cylindrical surface in the billet. The outer and inner radius of this layer, considering the final location of the tracked points from the simulation, were from 8 mm (the distance from the rolled billet’s center to point 3) to 16 mm (the distance from the center of the rolled billet to point 5).

**Figure 6:**
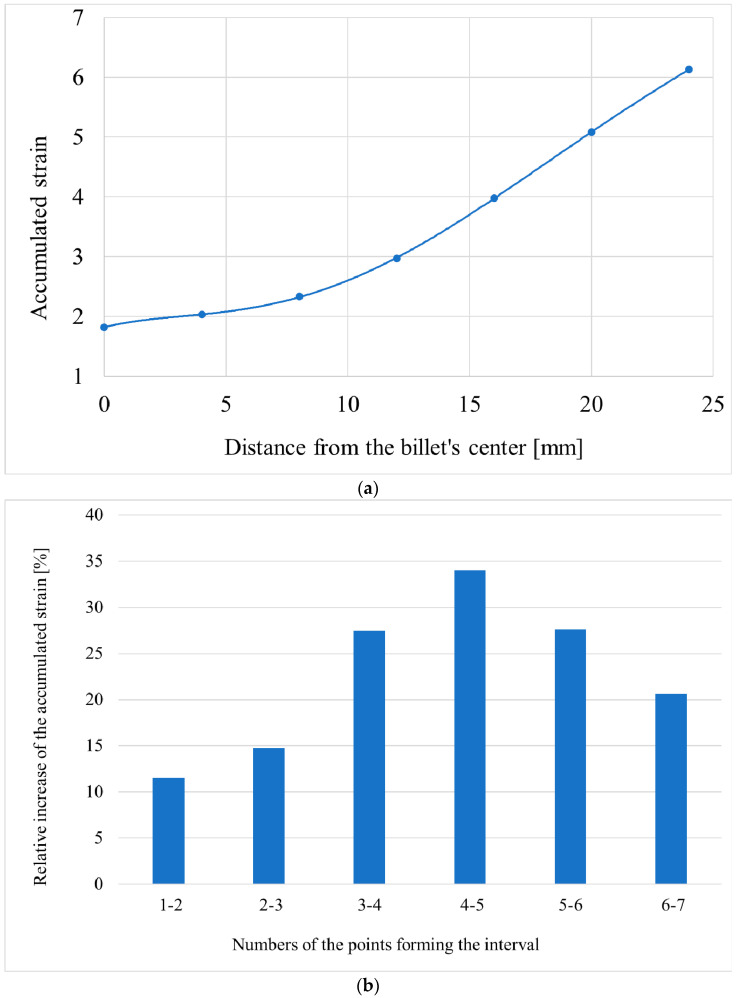
Variation in the accumulated strain along the rolled billet’s radius (**a**) and the relative increase in the accumulated strain in the intervals between tracked points (**b**) after computer simulation of all three passes.

The results of computer simulation made it possible to evaluate and illustrate, by means of a point diagram, how the accumulated strain value changed in the tracked points. Then, the trend line, which illustrated the accumulated strain variation along the billet’s radius, was calculated (Figure 6a). The relative increase (in percent) in the accumulated strain between the neighboring tracked points was also estimated (Figure 6b). 

Figure 6a shows that the accumulated strain value at the central region of the billet was three times lower that at the surface region. Hence, the surface microstructure should be characterized by a smaller grain size than the central region. Research dealing with the estimation of the microstructure after three-high RSR, for instance [40], found two areas that could be identified in the billet after RSR: peripheral with a fine-grain structure and central with more coarse grains. This is visible in Figure 6b, where the relative increase in the accumulated strain was positive, and then it was negative. Hence, the trend was the same as with the total velocity change, when one trend changed to another. With the accumulated strain, the positive relative increase was between points 4 and 5, that is, in the interval of 12–16 mm from the billet’s center.

The presence of the peripheral and central regions with different grain sizes implies that, in terms of statistics, the grain size distribution in the cross section of the billet should be formed by two distributions: one corresponding to the peripheral region and the other to the central region. There should be two clearly visible maximums (peaks) on the histogram. 

**Figure 7:**
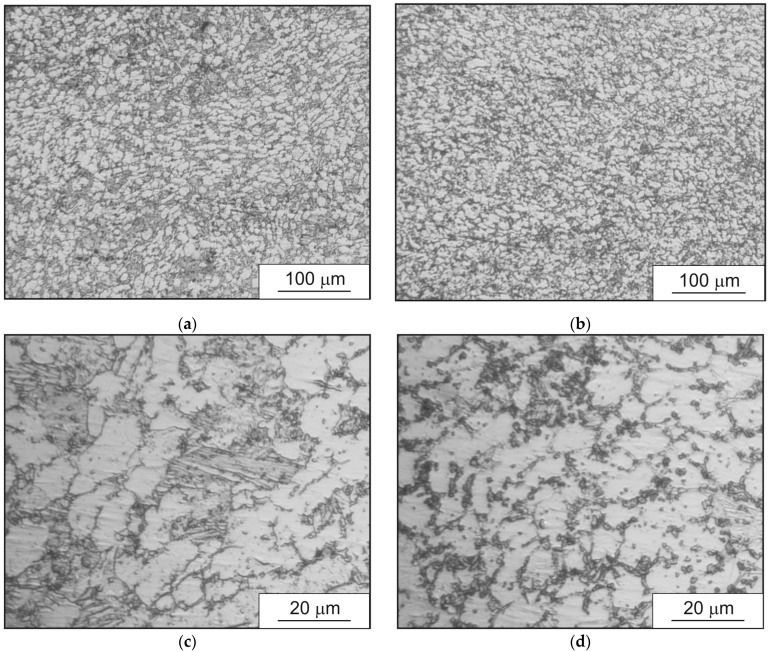
Ti-6Al-4V microstructure in the cross section of the billet: (**a**,**c**)—central region of the billet and (**b**,**d**)—near the surface region.

As a result of the research, the microstructure of the billet alloy after all three passes was estimated in the billet’s cross section (Figure 7).

According to Figure 7, there was dual-phase structure: bright grains of α-phase and laminar β-phase grains between α-phase grains. The grain size of the β-phase in the central region of the billet was up to 5 times higher than at the surface of the billet. This was consistent with the above discussion of the two layers in the billet, namely fine grains at the surface and coarse grains in the central region of the billet. The grain size of the α-phase in the center of the billet was slightly larger than at the surface. The microstructure (Figure 7) corresponded to the GOST 26492-85 named “Rolled bars from titanium and titanium alloys. Technical specifications” (Russian state standard).

**Figure 8:**
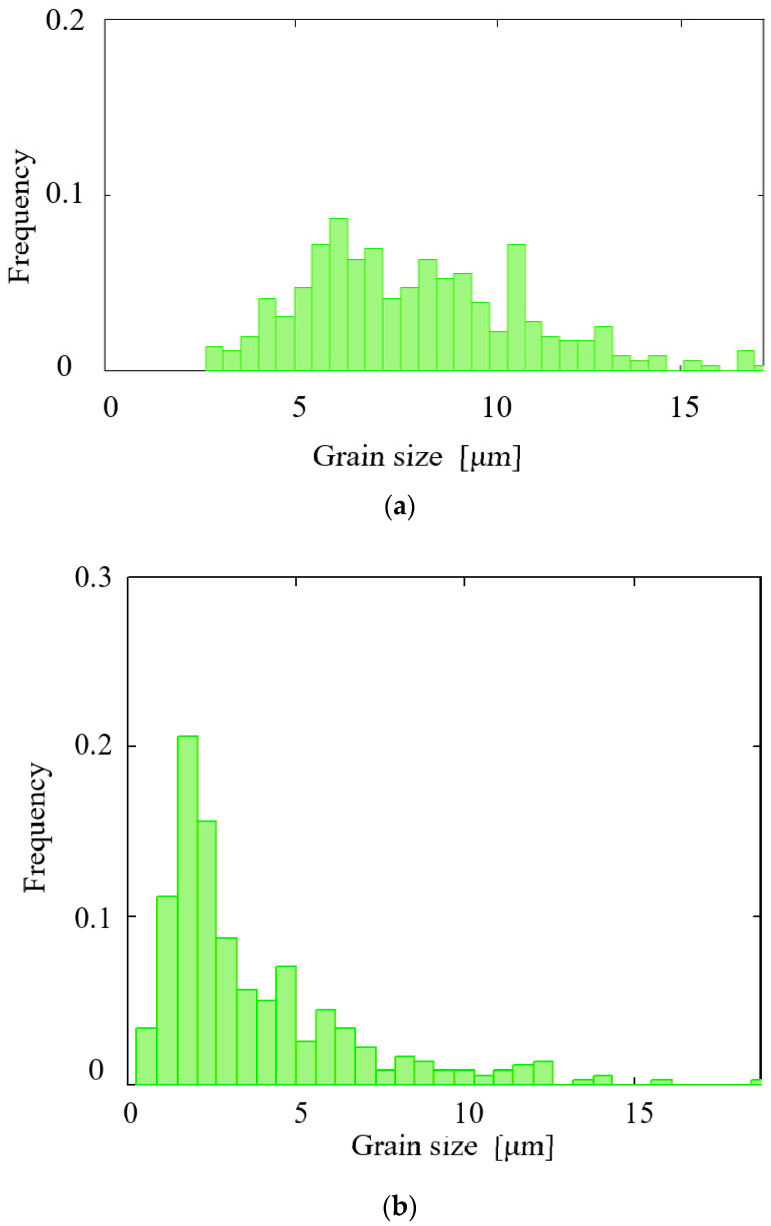
Histograms of the α-phase (**a**) and β-phase (**b**) grain size distribution in the cross section of the billet after radial shear rolling.

The histograms of the α-phase and β-phase grain size distribution in the cross section of the rolled billet (after all three passes) as a result of the microstructure research were plotted (Figure 8).

When plotting the histograms, different numbers of intervals were chosen (10 or more). The histograms had one or several maximums with frequencies 0.05 or less (as in Figure 8), or the histograms had only two maximums with frequencies 0.1 or more. The observed number of clearly visible columns (maximums) confirmed the above assumption that the grain size distribution was formed of two distributions.

**Figure 9:**
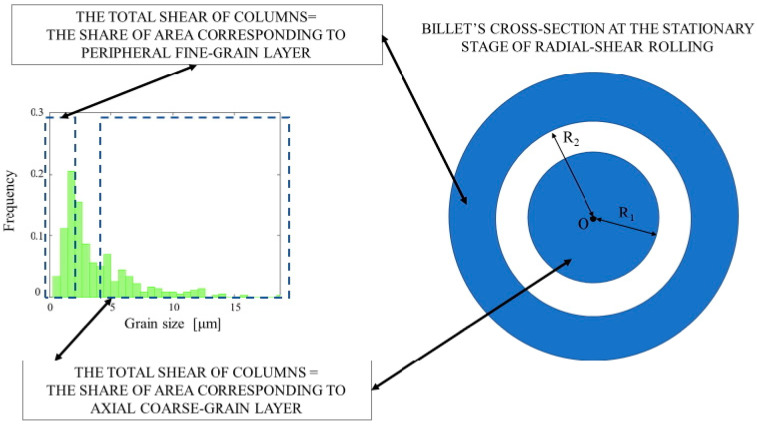
Detection of the borders of the region, which contains the neutral cylindrical layer based on the β-phase grain size distribution histogram analysis (O—center of the circle, which is the cross section of the billet at the stationary stage of the radial shear rolling, R_1_, R_2_—radii limiting the neutral layer).

A technique to determine the limits of the neutral cylindrical surface, whose radius range of variation was estimated using the computer simulation according to Figure 5 and Figure 6b, was designed. The technique was as follows. The left of the two highest columns (maximums) and the columns to left of it were identified (there were three columns in total). Their shares of all the grain sizes sampled were: 0.025, 0.110, and 0.205. The sum of the frequencies of all the specified columns was 0.340. This share (0.340) was set equal to the peripheral fine-grain layer share from the whole cross section area of the billet at the stationary stage of RSR (outer blue ring are on Figure 9). In an analogous way, the right of the two columns (maximums) was identified in the histogram along with the columns to the right of it. The shares of this right column (maximum) and the columns to the right of it in the sampling were calculated (there were 18 columns in total): 0.065, 0.020, 0.041, 0.031, 0.017, 0.005, 0.010, 0.009, 0.005, 0.004, 0.003, 0.005, 0.007, 0.008, 0.001, 0.002, 0.001, and 0.001. The total sum of these fractions was 0.235. This share (0.235) was set equal to the coarse-grain axial layer share from the whole cross section of the billet at the stationary stage (blue circle in Figure 9). With these shares’ values, using the circle formula, the values of the radii R_1_ and R_2_ were found; these radii corresponded to the border between the coarse-grain and fine-grain structures (Figure 9). The R_1_ was equal to 11.5 mm, and the R_2_ was equal to 19.5 mm. These radii constrained the neutral cylindrical layer, separating the peripheral coarse-grain and axial fine-grain layers. In other words, the radius of the neutral cylindrical surface, estimated by the β-phase grain size distribution histogram (Figure 8b) analysis, was within the 11.5–19.5 mm limits.

**Figure 10:**
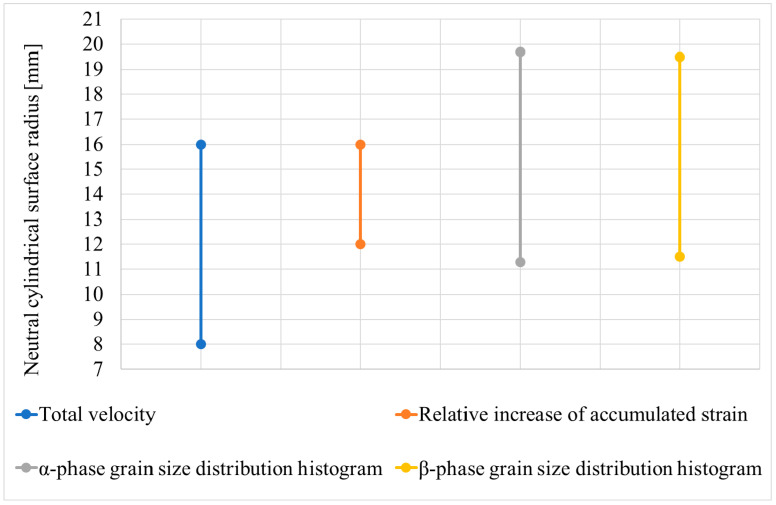
Limits containing the neutral cylindrical surface radius, which were calculated using the computer simulation and the experimental radial shear rolling.

According to the technique illustrated in Figure 9, the limits containing the neutral cylindrical surface radius were calculated using the α-phase grain size distribution histogram data (Figure 8a). The interval was 11.3–19.7 mm. The intervals limiting the neutral cylindrical surface radius were compared (Figure 10). Comparison was made for the limits calculated using the computer simulation results, namely the variation in the total velocity of the tracked points (Figure 5) and the relative increase in the accumulated strain (Figure 6b), and the limits calculated using the microstructure analysis, including the α-phase grain size distribution histogram (Figure 8a) and the β-phase grain size distribution histogram (Figure 8b) data.

When all data concerning the limits were joined, then 8–19.7 mm limits were obtained. When comparing the computer simulation and the experimental rolling results, the neutral surface radius interval calculated using the relative increase in the accumulated strain was entirely within the corresponding intervals calculated using the α-phase grain size distribution histogram and the β-phase grain size distribution histogram data. The neutral surface radius interval calculated using the variation in the tracked points’ total velocity while passing the draft was 57% within the corresponding interval calculated using the β-phase grain size distribution histogram data and 60% within the interval calculated using the α-phase grain size distribution histogram data. It is worth noting that the limits and, correspondingly, the sizes of the intervals within the computer simulation can be refined (decreased) by increasing the number of the tracked points lying on the billet’s radius.

The simultaneous analysis of the grain size distribution, the accumulated strain increment, and the total velocity change showed the presence of a neutral ring-shaped area at the three-high RSR; previously, none of these three items were used for such a purpose. The obtained results revealed the presence of a neutral layer in the shape of a ring and confirmed the results of previous studies revealing (directly or indirectly) a ring-shaped area [11,33,34,42,43]. The offered technique was less time consuming compared to those presented in [11]; plotting the total velocity graphs (Figure 5) as well as the accumulated strain increment diagram (Figure 6) was easier than creating the trajectories of [11], which was associated with the long processing of the FEM simulation results. Compared to using damage criteria [43], the designed technique does not needed to be readjusted when investigating other screw-rolling or RSR process regimes and other billet’s materials. As for further applications of the designed technique, it can be used for neutral layer dimensions’ estimation during three-high screw piercing. The concept of a ring-shaped area in the cross section of the billet is extremely important, as presented in [42]. The author of [42] clearly illustrated that the ring-shaped area was a key factor affecting hollow shells and seamless-tubes dimensions’ accuracy in three-high screw rolling. 

## 4. Conclusions

The computer simulation of radial shear rolling (RSR) was conducted using QForm software. The simulation was performed for the RSR of a Ti-6Al-4V alloy billet. The RSR was conducted in terms of rolling using an MISIS-100T mill in three passes, according to the route: Ø76 →Ø65→Ø55→Ø48 mm. Experimental rolling was performed according to the same route in an MISIS-100T rolling mill. 

It has been established, that:

1. The 48 mm diameter final billet had two regions in its cross section at the stationary stage, according to the computer simulation results: the outside (surface) region where the metal flow velocity decreased and the central (axial) region where the metal flow velocity increased. The neutral layer radius dividing these two regions was within 8–16 mm limits, i.e., 0.33–0.67 from the radius of the rolled billet.
2. The relative increase in the accumulated strain along the radius of the billet firstly increased monotonically and then decreased monotonically, according to the results of the computer simulation. The radii of the neutral cylindrical layer dividing the region with a monotonic increase in the accumulated strain from the region with monotonic decrease in the accumulated strain were within the 12–16 mm limits, i.e., 0.5–0.67 from the radius of the rolled billet.
3. The grain size distribution through the cross section of the billet was formed by the imposition of at least two homogeneous distributions according to the results of the microstructure research: the outer (surface) fine-grain region and the central (axial) coarse-grain region. Estimation of the neutral cylindrical surface inside the neutral layer, which divided these two regions (or layers) provided wider limits of 11.3–19.7 mm based on the α-phase research and 11.5–19.5 mm and a smoother transition between the layers based on the β-phase research.
4. The joint use of computer simulation and experimental estimation of microstructure enabled demonstrating that the microstructure formation during three-high RSR was caused by complex kinematic conditions and nonmonotonic variations in the kinematic and deformation parameters through the cross section of the billet. It is possible to regulate the dimensions, the ratio between size of the peripheral (surface) fine-grain layer and the axial (central) coarse-grain layer, and the sizes of structural elements inside each of the layers by varying the kinematic and deformation conditions of the RSR process.
5. The presence of a neutral layer in the shape of ring in the cross section of the billet during RSR was shown for the first time by analyzing three items simultaneously: the grain size histogram, the accumulated strain increment, and the total velocity change. The offered technique, compared to those previously designed, does not require any readjustment in the case of changing forming regimes, equipment setting, or the billet material; it also does not require a large amount of data processing after carrying out the FEM computer simulation.
6. The offered technique, without the grain size histogram, enables estimation of the neutral layer dimensions without any experimental rolling, through the FEM computer simulation. Considering that three-high screw rolling and RSR process are applied for forming of such expensive materials as titanium alloys, zirconium alloys, magnesium alloys, and high-alloyed steels, the designed technique could sufficiently reduce costs during screw-rolling and RSR process investigations.

## Figures and Tables

**Table 1:** Chemical composition of the Ti-6Al-4V alloy (in weight %).

Fe	C	Si	V	N	Ti	Al	Zr	O	H	Impurities
up to 0.6	up to 0.1	up to 0.1	3.5–5.3	up to 0.05	86.45–90.9	5.3–6.8	up to 0.3	up to 0.2	up to 0.015	0.3

## Data Availability

Not applicable.
